# A shielded 32‐channel body transceiver array with integrated electronics for 7 T

**DOI:** 10.1002/mrm.30498

**Published:** 2025-03-30

**Authors:** Tobey D. Haluptzok, Russell L. Lagore, Simon Schmidt, Gregory J. Metzger

**Affiliations:** ^1^ Center for Magnetic Resonance Research (CMRR) University of Minnesota Minneapolis Minnesota USA

**Keywords:** 7 T, body imaging, loop dipole, RF shield, transceiver array, UHF MRI

## Abstract

**Purpose:**

Develop a 32‐channel transceiver array for 7 T body imaging that incorporates an RF shield, improves SNR, lowers g‐factors, and is robust to external loading.

**Methods:**

The addition of a local RF shield was first investigated for single resonant blocks consisting of either one loop and a dipole (LD) or three loops and a dipole (3LD). A 32‐channel array consisting of eight shielded 3LD blocks (32LD‐SH) was constructed and validated for in‐vivo use. The SNR, parallel imaging, and transmit performance were compared to a previously published 16‐channel LD array (16LD). The effect of top loading was investigated by placing arms on top of the coils and measuring S‐parameter changes. In vivo imaging of multiple anatomies was performed.

**Results:**

In single block experiments, the RF shield impacted SNR and $B_{1}^{+}$ performance by <5%. The 3LD blocks had 80% higher peripheral SNR and 25% higher SNR at a depth of 10 cm. The 32LD‐SH array had 18% lower $B_{1}^{+}$/W^0.5^ efficiency and 30% higher central SNR compared to the 16LD array and supported threefold acceleration in the foot–head direction. Arm placement had no effect on the 32LD‐SH array but reduced the 16LD match to 5.4 dB.

**Conclusion:**

A 32‐channel transceiver array was developed for 7 T body imaging that is insensitive to top loading and has higher SNR and lower g‐factors compared to an existing 16‐channel transceiver array. Despite lower transmit performance, parallel transmit optimization permitted the 32LD‐SH to achieve flip angles necessary for high‐quality gradient and spin echo acquisitions of target organs in the chest, abdomen, and pelvis.

## 1 INTRODUCTION

Body imaging at ultrahigh fields (UHF) holds significant promise for improved performance in anatomical targets throughout the torso in both research and clinical settings.^1^, ^2^, ^3^ Advantages include increased SNR,^4^, ^5^, ^6^ enhanced parallel imaging performance,^4^, ^5^ and favorable relaxation properties. As in the brain, the presence of increased susceptibility contrast provides improved sensitivity to oxygenation^3^, ^6^, ^7^ and exogenous contrast media.^8^ Prolonged T_1_s lead to improvements in a host of applications, including in‐flow angiography and arterial spin labeling.^9^, ^10^, ^11^ To realize these advantages and achieve wider adoption, several technical developments are required, including improvements in RF coils and methods to manage the electromagnetic (EM) fields.

First, tailored RF management strategies that capitalize on state‐of‐the‐art parallel transmit (pTx) hardware are needed. Because the RF wavelength within tissue at 7 T (˜11 cm) is much shorter than the dimensions of the human torso, large transmit field inhomogeneities exist, necessitating the use of pTx methods. A whole host of improvements can be made in this regard, such as improved calibration data, tailored optimization strategies, and implementation of acquisition methods incorporating dynamic pTx pulses.

Second, robust RF coils that provide optimal pTx performance and capture the expected SNR gains for applications throughout the human torso are needed. Although there is literature on the development of in‐bore transmitters at 7 T,^12^, ^13^ most RF coils for UHF body imaging focus on more closely fitted transmit and receive (Tx/Rx) arrays due to existing RF architectures and cost. Advances in antenna‐based RF coil designs for body imaging have included dipole elements that have demonstrated superior SNR,^14^ increased peak specific absorption rate (pSAR) efficiency,^15^, ^16^, ^17^, ^18^ and current distributions closely approximating the theoretical optimum.^19^ Moreover, their inherent geometric decoupling facilitates integration with conventional surface loop elements.^20^, ^21^, ^22^ Although exhibiting high Tx/Rx efficiency, local RF coils are susceptible to variable loading conditions.^23^ In some instances, even minor changes such as placing an arm on top of a coil element, which we will refer to as *top loading*, can alter the coil's tuning and matching enough to impact SNR, invalidate safety factors of carefully characterized coils, and negatively impact the reliability of real‐time power monitoring.

In this paper, we present a new shielded 32‐channel transceiver array consisting of eight blocks, with each block being comprised of three geometrically decoupled loops and a fractionated dipole. An important component of this design is an RF shield that makes the array insensitive to top loading, simplifies cable management, and enables integrated on‐coil electronics. We compare its performance to a previously presented unshielded 16‐channel transceiver array consisting of eight single‐loop dipole blocks from Ertürk et al.^20^ Although transmit performance decreased, we show that the new design has an increased SNR, has better parallel imaging performance, is significantly less sensitive to top loading, and maintains transmit efficiency sufficient for gradient echo (GRE) and turbo spin echo (TSE) imaging in multiple anatomies.

## 2 METHODS

A 32‐channel array was developed in a multi‐step process. The initial design, adapted from a single‐loop dipole transceiver array,^20^ involved splitting each loop into three loops, with the initial goal being enhanced Tx/Rx performance guided by previous simulation results.^24^ Subsequently, an RF shield was incorporated into the design to facilitate on‐coil electronics and minimize the impact of top loading. Finally, all coil electronics, including preamplifiers (preamps) and Tx/Rx switches, were implemented behind the RF shield. This simplified cable management and reduced the overall receiver noise figure. The following sections detail the design, implementation, and evaluation of each developmental stage, first as single blocks and then as a complete array.

### 2.1 Single block design

Initially, four distinct block configurations were designed and tested. Each block incorporated a fractionated dipole element matched via a lattice balun. The first configuration, the loop‐dipole (LD), combined the dipole with a single rectangular loop (18 × 8 cm^2^) featuring five capacitor tuning breaks. An L‐match network with a symmetric virtual ground was employed to minimize common‐mode currents and cable interactions. The second configuration, the three‐loop dipole (3LD), integrated the dipole with three overlapping loops arranged to maintain an axial length similar to the LD.^24^ The 8 × 8 cm^2^ loops used 6 mm wide conductors with 12 mm overlaps for nearest‐neighbor decoupling^25^ and four distributed capacitors. The third and fourth configurations were shielded versions of both the LD and 3LD (LD‐SH and 3LD‐SH). Because the RF shield created different loading conditions, the shielded elements required slightly different tuning and matching.

All dipole and loop elements were fabricated on 1.57 mm thick FR4 printed circuit boards. Nonmagnetic ceramic chip capacitors were used for tuning and matching. In all cases, the loops were centered along the dipole's long axis to minimize coupling. For assembly, the resonant elements were mounted on top of a 5 mm thick polylactic acid spacer, whereas a 20 mm thick polylactic acid spacer was used to position the RF shield above the resonant elements.

### 2.2 The RF shield

**FIGURE 1:**
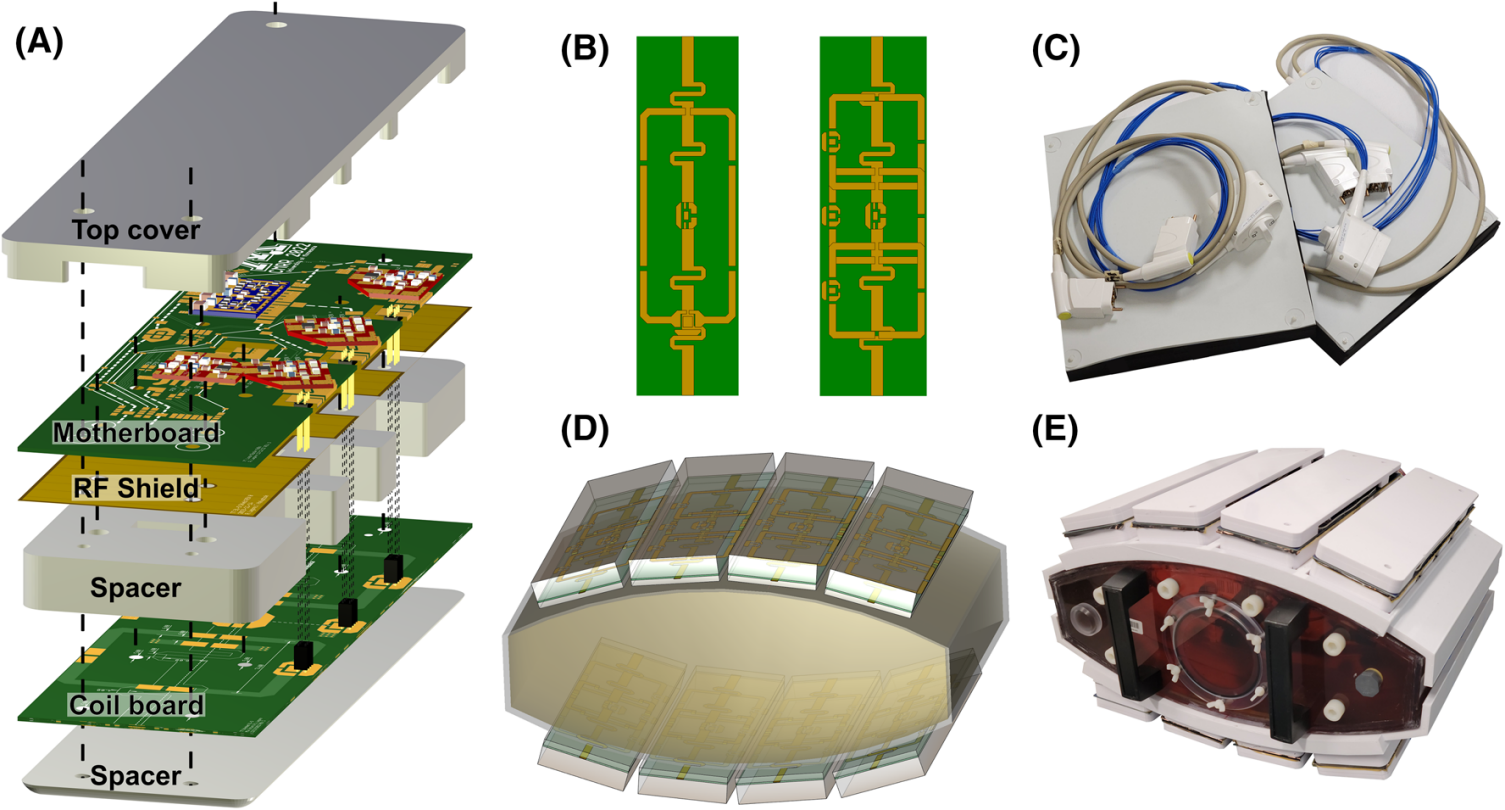
Images showing the rendering of (A) a complete 3LD‐SH block, (B) the individual LD and 3LD elements, (C) the final coil array in a foam housing, (D) the simulated array for coil validation, and (E) the physical coil array on a human body phantom. 3LD, three loops and a dipole; LD, one loop and a dipole; SH, shielded.

Integrating copper shielding into each block posed several challenges but also offered multiple benefits. These benefits include: (1) reduced sensitivity to top loading; (2) simplification of coil simulations by eliminating cable routing effects; and (3) creation of an EM shadow, allowing for easier cable management and on‐coil electronic placement without compromising transceiver performance. The RF shield implemented in these experiments had penetrations for RF interconnects (Figure 1A), which simplified cable routing. Ideally, cables are routed along virtual ground paths to minimize cable interactions and are perpendicularly attached to coil elements. However, this is often impractical to implement. With the proposed design, interconnects are naturally routed perpendicular to the resonant elements with minimal cabling being present between the coil elements and the RF shield, thereby minimizing cable interactions. One of the main concerns with RF shielding is its potential impact on coil performance, which is rigorously evaluated in the subsequent single block comparison. Additionally, eddy current formation can degrade image quality. To mitigate eddy currents,^26^ the RF shield was constructed using the thinnest commonly available double‐sided flexible printed circuit laminate from PCBWay (Hong Kong, CN). This laminate features a copper thickness of 12 μm and a polyimide dielectric thickness of 25 μm. The two layers were segmented into 10 mm wide strips with 0.1 mm gaps separating neighboring strips. The gaps on one side aligned with the center lines for segments on the other side, effectively creating a continuous RF shield through capacitive coupling between overlapping strips.^27^ To ensure that segmenting the RF shield does not affect performance, its Q ratio was compared against a solid copper shield by measuring with two decoupled H field probes. The unloaded/loaded Q was 200/18 for the solid copper shield and 175/18 for the segmented shield, resulting in Q ratios of approximately 11 and 10, respectively. Single block SNR and $B_{1}^{+}$ experiments were also performed and no measurable differences between the two shield configurations was observed.

### 2.3 Single block comparison

In this evaluation, the $B_{1}^{+}$ field was compared with the assumptions that each element on the block was driven with an independent transmitter and that the total forward power to each block was equal. Experimentally, all blocks were connected to the same remote interface box that housed Tx/Rx switches, allowing us to concentrate on evaluating the different coil geometries and the presence of the RF shield.

#### 2.3.1 Simulation

The Tx/Rx performance of the blocks were assessed numerically using Sim4life 7.2 (ZMT Zurich MedTech AG, Zurich, Switzerland). Each block model was positioned 18 mm from a human body‐sized phantom consisting of a human tissue mimicking polyvinylpyrrolidone solution (relative permittivity ε_r_ = 51.4, electrical conductivity σ = 0.57 S/m at 297 MHz). The conductors and lumped elements of the blocks were meshed at 1 mm resolution, whereas the remainder of the model used an automatically calculated rectilinear grid with a maximum size of 5 mm. A Gaussian excitation profile centered at 297 MHz with a 100 MHz bandwidth was used in a finite‐difference time‐domain solver to generate S‐parameter curves. Lumped elements were tuned to achieve ≥15 dB matching at 297 MHz. The resulting EM field distributions at 297 MHz were normalized to 1 W accepted power and exported to mat files for calculating intrinsic SNR, $B_{1}^{+}$ power efficiency ($\eta_{p}$, μT/ W^0.5^), and $B_{1}^{+}$ SAR efficiency ($\eta_{\mathrm{SAR}}$, μT/pSAR^0.5^). Intrinsic SNR was defined as the thermal noise decorrelated $B_{1}^{-}$ field, as described in previous works.^19^, ^28^, ^29^, ^30^ For the LD test blocks, we used a 1/2 W power split to the loop and dipole in this analysis. For the 3LD blocks, we used 1/6 W and 1/2 W to each loop and dipole, respectively, mimicking the three‐way power divider used in the physical array implementation discussed below. A phase‐only RF shim maximizing transmit efficiency was calculated using the MatLab 2024a (MathWorks, Natick, MA) *fmincon* function to solve the minimization problem given in Equation 1, where $N_{v}$ is the set of all voxels in the phantom, $N_{c}$ is the number of coil channels, x is the phase‐only shim vector, and $B_{1}^{+}$ is the complex channel‐wise transmit map.

(1) $$\min_{x}-\frac{\sum_{N_{v}}|x\cdot {B_{1}}^{+}|}{\sum_{N_{v},N_{c}}\left|{B_{1}}^{+}\right|}.$$

The resulting Tx/Rx performances are shown in absolute and relative maps and quantitatively analyzed with violin plots at varying depths into the phantom with respect to SNR, $\eta_{p}$, and $\eta_{\mathrm{SAR}}$.

#### 2.3.2 Experiments

Because each block in this comparison had multiple channels, relative $B_{1}^{+}$ maps were acquired^31^ and used to calculate the phase set applied during absolute $B_{1}^{+}$ mapping. Individual coil block absolute $B_{1}^{+}$ maps were measured using the actual flip angle imaging technique^32^ on a whole‐body Magnetom 7 T scanner (Siemens Healthineers, Erlangen, Germany) using the same remote Tx/Rx switch box (Virtumed, Minneapolis, MN) and a physical version of the body phantom used in the simulations given above (Figure 1E). MRI acquisition parameters can be found in Table S1.

### 2.4 Array design

A shielded 32‐element array (32LD‐SH) was constructed using eight 3LD‐SH blocks (Figure 1E). Capitalizing on the presence of the RF shield, all active electronics were placed directly on the block, behind the shield, on a custom‐designed motherboard (Figure 1A). Each motherboard integrated four Tx/Rx switches equipped with low‐noise amplifiers (QPL9547, Qorvo Inc., Greensboro, SC), DC power lines for active PIN diode switched preamp protection, rectangular six‐pin RF interconnects (SAM1060‐03‐ND, SAM1214‐03‐ND, Samtec, New Albany, IN), and a three‐way Wilkinson power divider. All Tx/Rx switches had less than a 0.35 dB insertion loss, a worst‐case return loss of 1.25 dB, and provided at least 40 dB of preamp protection during transmit. The preamp has a 50 ohm impedance, a 0.3 dB noise figure, and 26 dB gain. No preamp decoupling is utilized in this array. The power divider was designed and built in‐house using lumped element quarter‐wavelength low‐pass Pi‐networks and a delta configuration of isolation resistors. Circuit schematics and pictures of example boards are given in Figure S1, and component values are given in Table S2. A fixed relative phase between the loops of 0 degrees was empirically determined, implemented in hardware using coax phase shifters, and experimentally validated to result in a desirable $B_{1}^{+}$ field. This new array was compared to an existing array comprised of eight LD blocks from above, as previously published.^20^

For the 32LD‐SH, transmit cables consisted of two bundles, each having eight low‐loss coaxial cables (Multiflex_86, Huber + Suhner Inc., Herisau, Switzerland), and terminated in an eight‐channel system plug (ODU GmbH & Co. KG, Mühldorf am Inn, Germany). One plug is dedicated to the anterior blocks, whereas the other services the posterior. Receiver cabling consisted of four 1Tx/8Rx TIM plug assemblies (Total Imaging Matrix, Siemens Healthineers). Each assembly supplied two blocks, grouped into anterior left, anterior right, posterior left, and posterior right (Figure 1C).

### 2.5 Full array comparison

A custom holder was designed to position individual array blocks 13 mm from the body phantom and 1 cm apart (Figure 1E). Following the validation procedure described by Schmidt et al.,^33^ a CT scan of the 32LD‐SH array and holder mounted on the phantom was used to create a 3D model for accurate block placement in simulation.

#### 2.5.1 Simulation

The 32LD‐SH and 16LD arrays were simulated on the body phantom and the Duke virtual human body model (IT'IS Foundation, Zurich, Switzerland) in three configurations with the array centered over the prostate, kidneys, and heart at a distance of at least 18 mm to the skin of the body model. The same meshing parameters were used in all simulations, with the conductors and lumped elements meshed with 1 mm resolution. All other entities were meshed at 2 mm. The resulting 1 W accepted channel‐wise EM fields were exported to MatLab (MathWorks) for further analysis. Randomly generated shims (*n* = 1000), along with a circularly polarized (CP) shim, defined as a 90‐degree phase between the loops and dipole and a 45‐degree increment between blocks, were applied to the simulated and experimental $B_{1}^{+}$ maps. The 99.9th percentile voxel‐wise $B_{1}^{+}$ error of the CP mode was taken to be the simulation error because this error was higher than the 99.9th percentile error from the 1000 random shims.^34^ To determine the performance impact when the loops of each block are powered with a single transmit channel, the full 32LD‐SH array was simulated in a 16‐channel Tx configuration (32LD‐SH [16Tx]) by applying the same 0‐phase, 1:3 power split implemented in the physical array. Static pTx performance was determined by solving the pSAR‐regularized coefficient of variation (CV) minimization problem as described in Equation 2, using virtual observation points^35^, ^36^ for efficient SAR calculations.

(2) $$\min_{x}\left[\frac{\sigma \left(x\cdot {B_{1}}^{+}\right)}{\bar{|x\cdot {B_{1}}^{+}|}}+\lambda *\frac{\sqrt{\max\left(x^{H}\cdot \mathrm{VOP}\cdot x\right)}}{\bar{|x\cdot {B_{1}}^{+}|}}\right].$$

Here, **x** is the complex shimming vector, σ denotes the SD in a defined volume of interest (VOI), and $\bar{\left|x*{B_{1}}^{+}\right|}$ the mean in the same VOI. The VOI was either the heart, kidneys, or prostate, depending on the position of the RF coil in the respective simulation.

The regularization parameter λ balances CV and pSAR, generating an L‐curve to illustrate the trade‐off between achievable B_1_^+^ homogeneity and the pSAR constraint.

#### 2.5.2 Phantom experiments

To evaluate the effects of local preamps and the RF shield on coil performance, the 32LD‐SH(16Tx) and 16LD arrays were positioned on the same phantom as in the single block comparison, and absolute $B_{1}^{+}$, SNR, and g‐factor maps were determined. Absolute $B_{1}^{+}$ and SNR scans followed the single block protocol, whereas g‐factor maps were derived from fully sampled SNR scans through retrospective undersampling, varying the acceleration factors and directions. SNR was also measured at 3 T (Magnetom Prisma, Siemens Healthineers) using the same acquisition strategy. At 3 T, the standard in‐bore body coil was used for transmit, and the 32‐channel spine and 18‐channel body arrays were used for receive.

#### 2.5.3 RF shield performance to top loading

To assess the influence of RF shielding on topload sensitivity, the anterior sections of the 32LD‐SH and 16LD arrays were connected to a 16‐channel vector network analyzer (Rohde & Schwarz, Munich, Germany) and positioned over the kidneys of a volunteer. S‐parameters were measured with the volunteer's arms positioned both at their side and atop the coil.

#### 2.5.4 In vivo experiments

To demonstrate the imaging performance of the 32LD‐SH coil, six individuals were imaged under an institutional review board–approved protocol. Before imaging, volunteers provided signed written consent after being medically screened and cleared to participate in the studies. Imaging acquisitions suitable for exploring multiple anatomies were performed targeting the prostate, hips, kidneys, liver, and heart. In the prostate, transverse full FOV TSE images were acquired using two acquisition modes optimized for refocused echoes (AMORE) modes.^37^ Localized coronal TSE imaging of the prostate was acquired with an efficiency shim.^38^ Anatomic imaging of the pelvis was acquired using a multi‐echo, water‐selective,^8^ 3D GRE that utilized two AMORE modes. Vessel imaging in the kidneys was acquired using a sequential 2D fat‐suppressed GRE. Anatomical imaging of the kidneys was acquired with both TSE and fat‐suppressed GRE acquisitions. All kidney images were acquired with the same efficiency shim calculated over both kidneys and in a single breath‐hold. Anatomical liver imaging was acquired with a 2D multi‐slice GRE using two AMORE modes. Cardiac CINE was performed with a retrospectively gated GRE using a peripheral pulse waveform obtained with the MRI system's physiological monitoring unit. Standard cardiac views were acquired, including the four‐chamber and short‐axis, each reconstructed with 50 phases. RF shimming for the cardiac scans consisted of a phase‐magnitude solution minimizing the CV with SAR constraints. Every AMORE scan along with the cardiac scans were bias‐corrected^39^ to highlight contrast homogeneity. Scan parameters for all acquisitions can be found in Table S1.

## 3 RESULTS

### 3.1 Single block comparison

**FIGURE 2:**
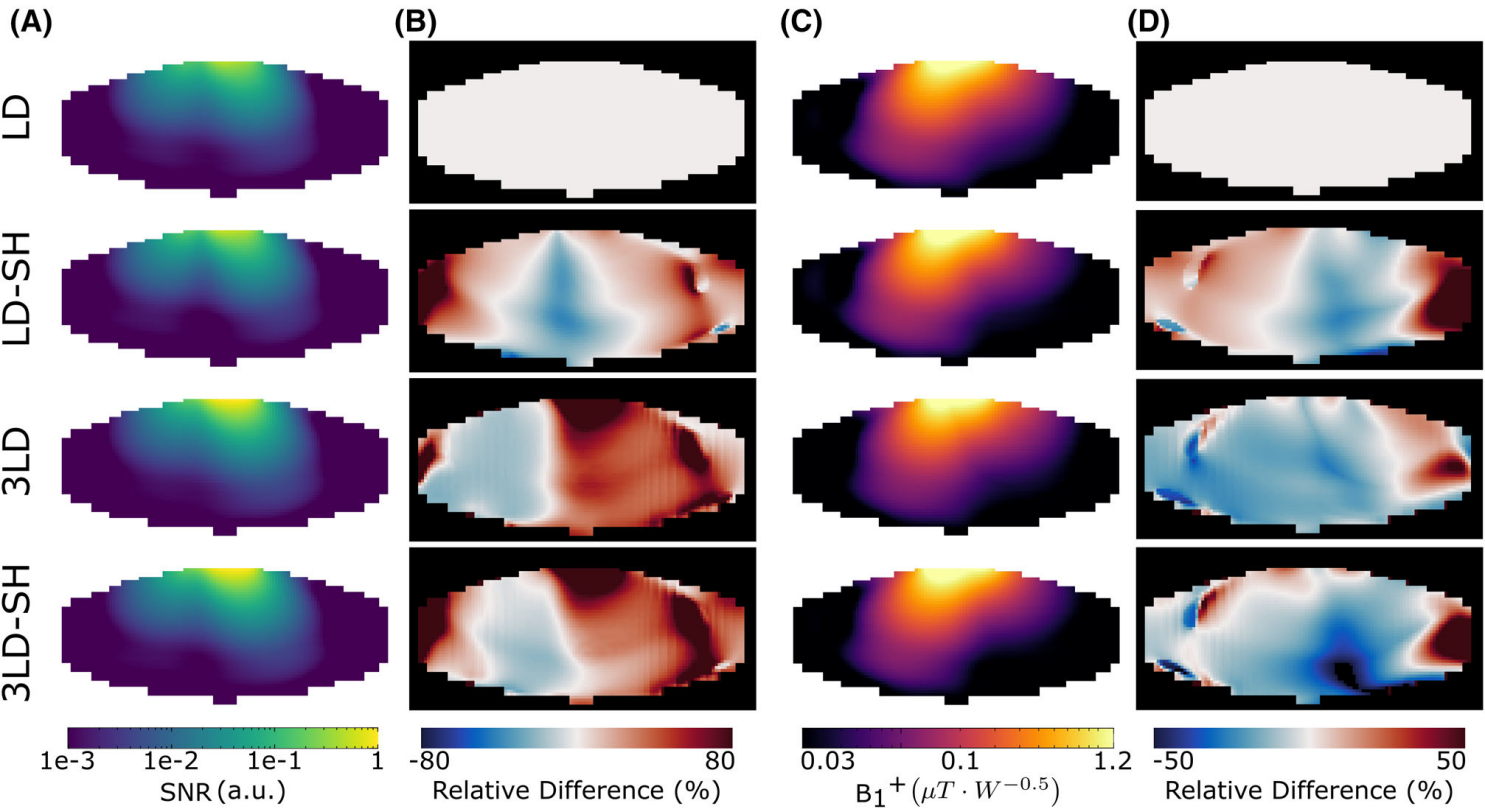
Images showing (A) simulated SNR and (C) $B_{1}^{+}/\sqrt{W}$ maps for the single‐coil block comparison. Both the SNR and $B_{1}^{+}/\sqrt{W}$ maps are shown on a logarithmic scale for better visualization. To get a qualitative comparison between the blocks, normalized difference maps are shown for (B) SNR performance and (D) $B_{1}^{+}/\sqrt{W}$ performance for each coil with respect to the LD coil.

**FIGURE 3:**
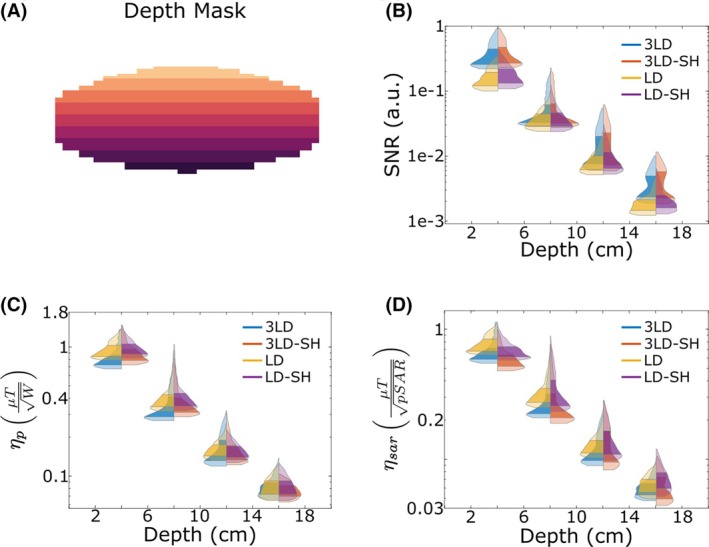
An analysis of simulated single block performance. Quantitative violin plots showing the top 20th percentile voxels at different depths into the phantom. The mask in (A) shows the regions of the phantom used to calculate (B) the violin plots at each depth for the SNR, (C) $\eta_{p}$, and (D) $\eta_{\mathrm{sar}}$ metrics. Although this is shown as a 2D slice, the actual mask includes all values along the Z axis of the phantom.

Simulated SNR and phase‐only efficiency shimmed $B_{1}^{+}$ maps for the four different coil block configurations are shown in Figure 2, along with relative difference maps compared to the reference unshielded LD block. The quantitative Rx/Tx performance of the four blocks at varying depths in the phantom are shown in Figure 3. Each depth is a 2 cm thick slice into the phantom, and the violin plots display the top 20th percentile pixels at each depth as a histogram. For increased readability, the violin plots are presented at depths of 2–4, 6–8, 10–12, and 14–16 cm.

The relative SNR maps in Figure 2D show that the 3LD and 3LD‐SH blocks outperform the LD blocks. This is shown quantitatively in Figure 3B as a function of depth. Compared to the LD blocks, the 3LD blocks exhibited 80% higher SNR in the periphery and 25% higher SNR 10 cm into the phantom. Importantly, the shielded and unshielded blocks had negligible performance differences.

On the transmit side, LD blocks outperformed the 3LD blocks (Figure 2A,B). The 3LD and 3LD‐SH blocks had similar $\eta_{p}$, with the 3LD‐SH block performing approximately 3% better over the different depths (Figure 3C). Conversely, the 3LD demonstrated higher pSAR efficiency, outperforming the 3LD‐SH block by approximately 5% (Figure 3D).

When quantifying transmit performance, an RF shimming solution was needed to compute a single combined $B_{1}^{+}$ field. A whole‐volume efficiency shim resulted in a $B_{1}^{+}$ field that realized 82% of the available $B_{1}^{+}$ in the entire left half of the phantom, where the main transmit lobe is positioned, and 50% of the available $B_{1}^{+}$ in the minor lobe (Figure S2). The optimal shim for the 3LD‐SH had relative loop phases of 0, −8, and −9 degrees, which is in close agreement with the empirically determined 0‐degree phase solution implemented on the full array. Experimental transmit efficiency results (Figure S3) showed close agreement with simulation. Considering the superior SNR of the 3LD‐SH block compared to the reference LD block, similar transmit performance relative to the unshielded 3LD block, and the benefits that come from the addition of shielding, a full array consisting of eight 3LD‐SH blocks was constructed.

### 3.2 Full array comparison

#### 3.2.1 Receive performance

**FIGURE 4:**
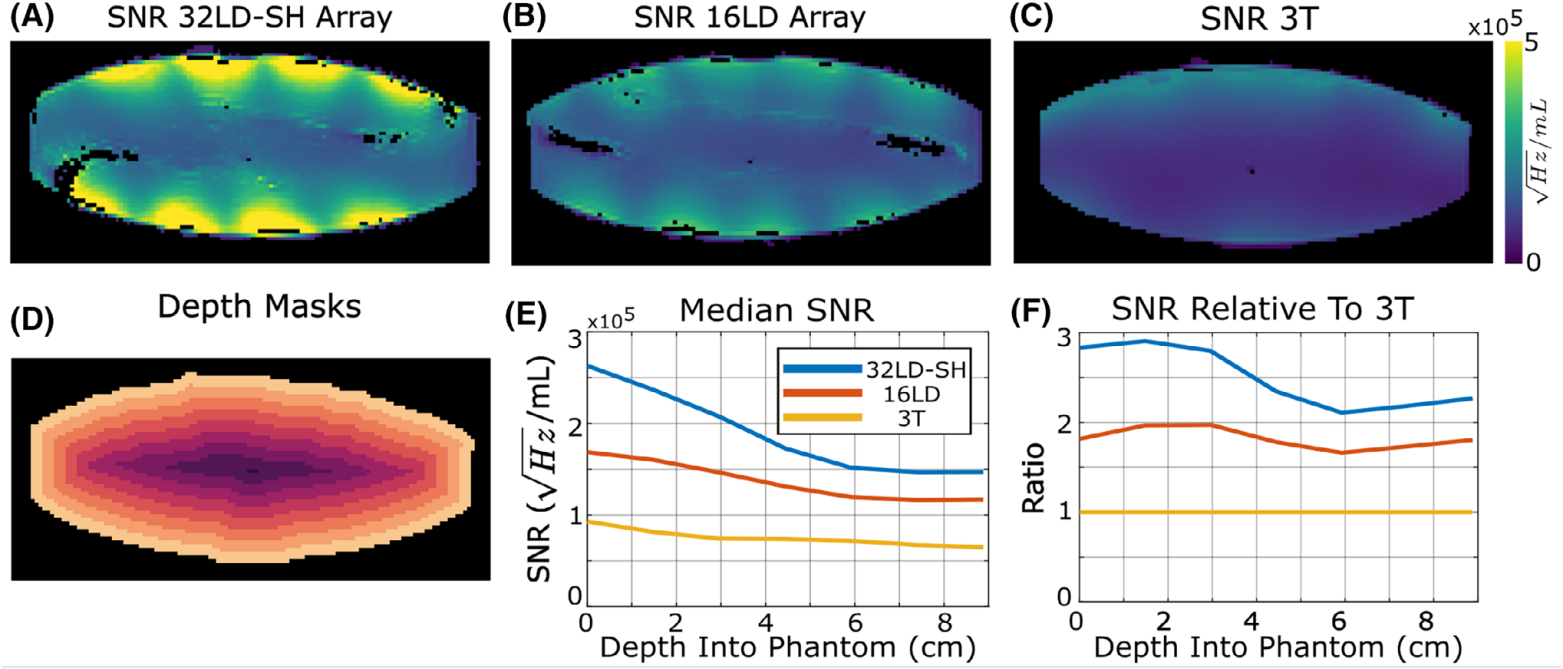
Experimental SNR results when scanning the body phantom with (A) the 32LD‐SH, and (B) the 16LD arrays at 7 T, and with (C) a standard imaging setup at 3 T. To get a quantitative SNR comparison, the median SNR at different depths (D) into the phantom was calculated. Absolute maps are shown in (E) and relative performance in (F)

The 32LD‐SH array demonstrated superior SNR performance at all depths compared to the 16LD array (Figure 4). On the surface and in the center of the phantom, the 32LD‐SH array achieved 80% and 30% higher SNR, respectively. Both 7 T arrays outperformed a standard 3 T setup, with the 32LD‐SH array having a 225% improvement in central SNR and 290% improvement in peripheral SNR compared to the 3 T receiver array.

#### 3.2.2 Parallel imaging performance

**FIGURE 5:**
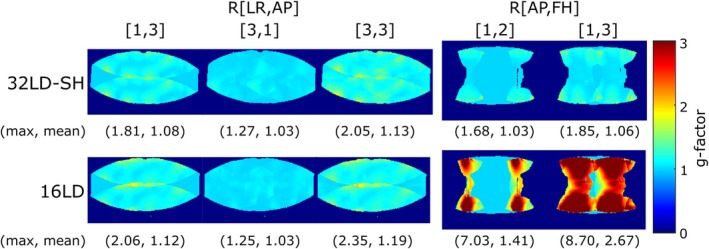
Experimental g‐factor maps calculated from retrospective under sampling of the SNR scan data where each map is a maximum intensity projection along the unaccelerated dimension. The top row shows the 32LD‐SH, and the bottom row shows the 16LD parallel imaging performance. The leftmost group of g‐factor maps shows accelerations of *R* = 3 in the LR and AP directions, whereas the rightmost group shows g‐factor maps when accelerating in the FH direction. AP, anteroposterior; FH, foot–head; LR, left–right.

G‐factor maps for the 32LD‐SH and 16LD arrays are shown in Figure 5. Both arrays performed similarly in the left–right and anterior‐posterior directions, enabling 3 × 3 2D acceleration while maintaining maximum and mean g‐factors below 2.5 and 1.25, respectively. However, when the acceleration direction is switched to the foot–head direction, the 32LD‐SH array maintains g‐factors with a mean value below 1.1 for a threefold acceleration, whereas the 16LD array encounters elevated g‐factors with a mean of 2.67.

#### 3.2.3 32LD‐SH(16Tx) validation

**FIGURE 6:**
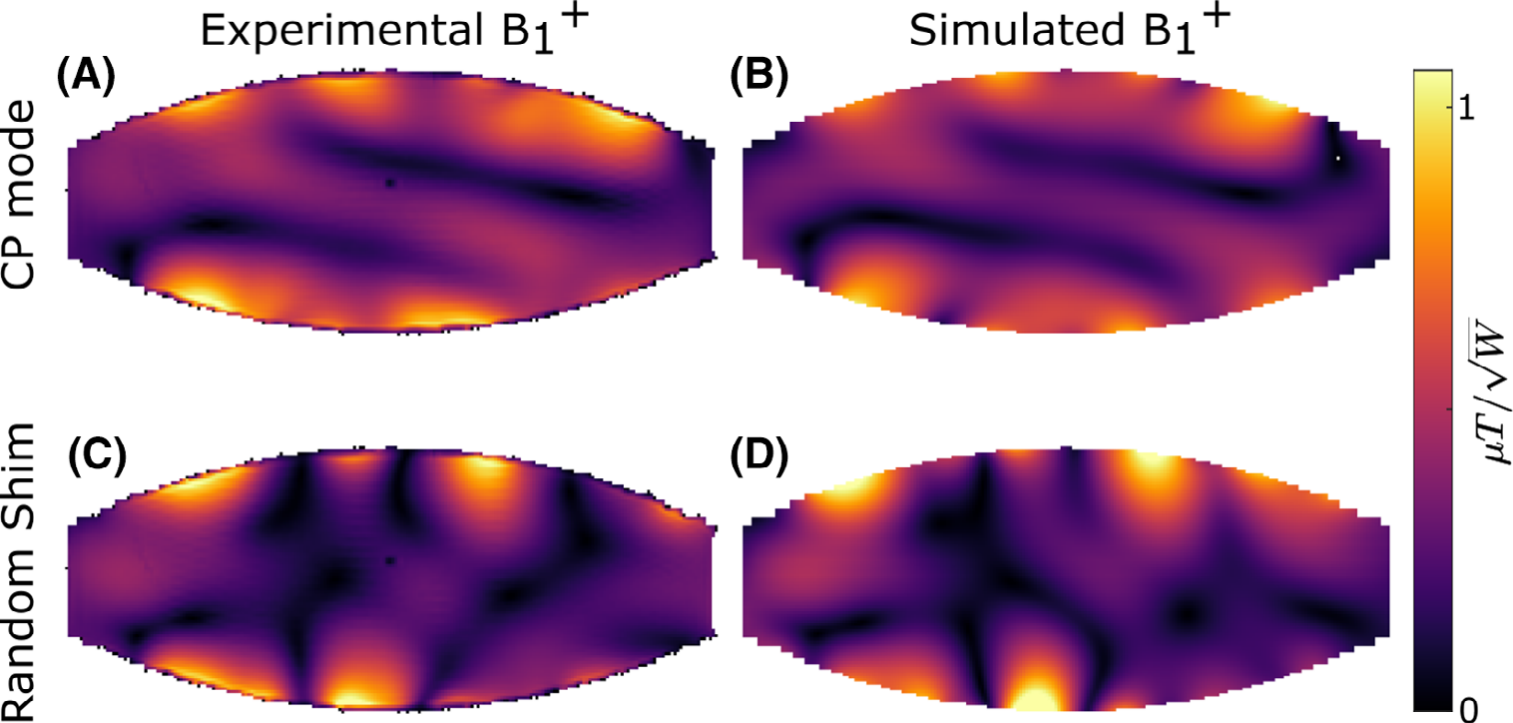
Experimental absolute B_1_^+^ from the 32LD‐SH array with (A) a CP mode excitation and (B) the predicted CP mode B_1_^+^ from simulation. Additionally, B_1_^+^ maps were compared using a random phase‐only shim applied (C) experimentally as well as (D) in simulation. CP, circularly polarized

Simulated and experimental $B_{1}^{+}$ maps for the 32LD‐SH(16Tx) array are depicted in Figure 6. The 99.9th percentile relative B_1_^+^ difference^34^ of 31% from the CP shim was used to define the modeling error. Combined with a 15% power monitoring error, specified by the vendor, and the previously determined 85%, 121%, and 141% intersubject variability in the pelvis, kidneys, and heart respectively,^33^ final safety factors of 192%, 226%, and 245% were applied to the 10 g averaged Q‐matrices calculated for Duke. Subsequently, these scaled Q‐matrices were compressed^36^ with a 10% overestimation error to create three sets of virtual observation points of size 136, 156, and183 for the pelvis, kidneys, and heart.

#### 3.2.4 Transmit performance

**FIGURE 7:**
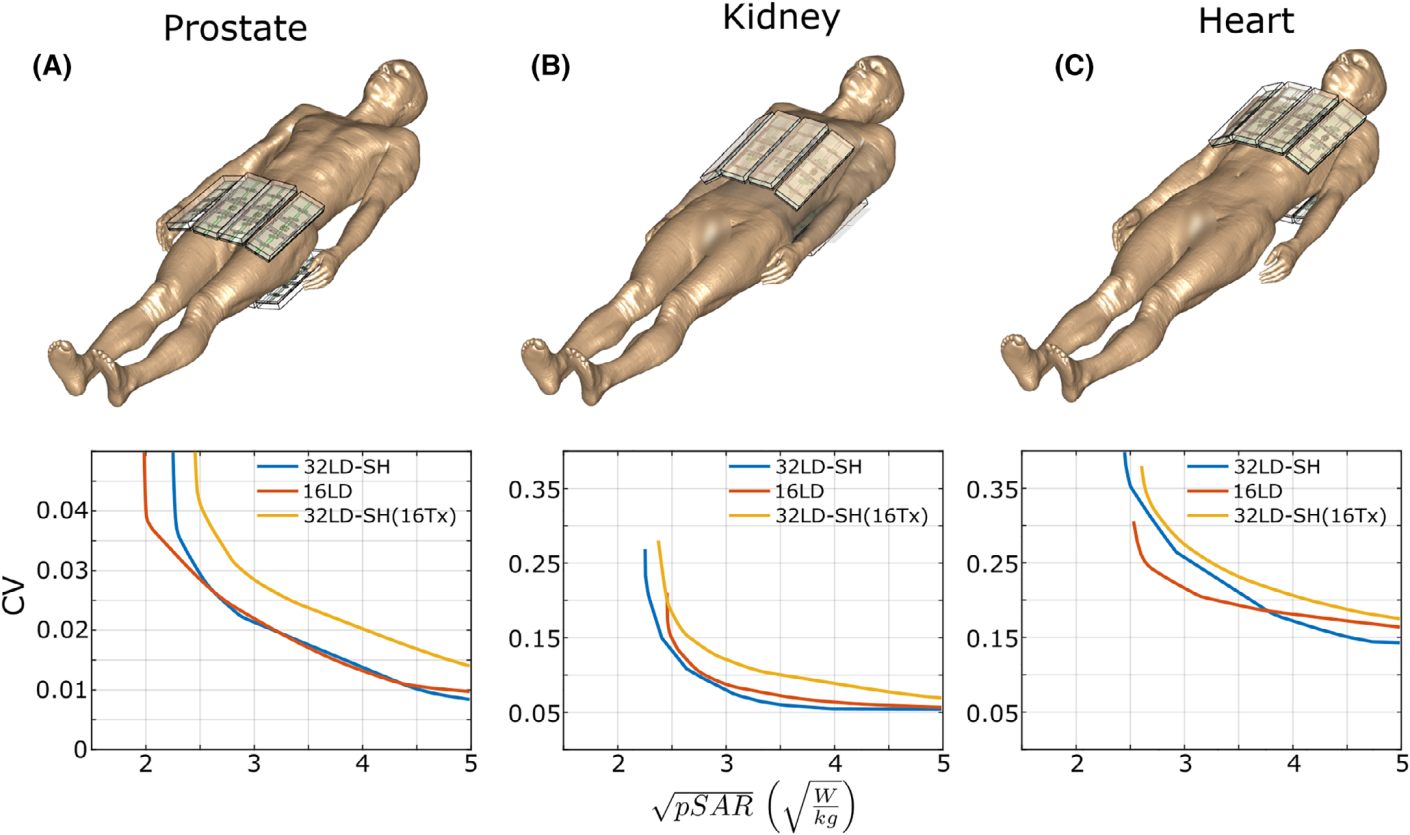
L‐curve analysis for the 32LD‐SH(32Tx), 32LD‐SH(16Tx), and 16LD. The first column (A) shows results when the arrays are placed over the prostate, the middle column (B) for the kidneys, and the right column (C) for the heart. The root pSAR is normalized to maintain the same mean B_1_^+^ in the target anatomies for every shim and is therefore the inverse of B_1_^+^ SAR efficiency $\eta_{\mathrm{sar}}$. Note that the CV scale is different in (A) to preserve the dynamic range. CV, coefficient of variation; pSAR, peak specific absorption rate; SAR, specific absorption rate; Tx, transmit.

The tradeoff between $\eta_{\mathrm{sar}}$ and B_1_^+^ CV is shown in Figure 7 as an L‐curve for three different target anatomies. Achieving uniform flip angles necessitates higher power and pSAR to maintain a consistent mean B_1_^+^. Conversely, tolerating increased inhomogeneity allows for enhanced $\eta_{\mathrm{sar}}$, culminating in the highest efficiency when there is no CV regularization. The L‐curves of the 32LD‐SH, 32LD‐SH(16Tx), and 16LD reveal that the 32LD‐SH(16Tx) exhibited performance marginally worse than the 32LD‐SH, having 9% worse $\eta_{\mathrm{sar}}$ in the prostate, 7% more pSAR to achieve a CV of 0.15 in the kidneys, and 8% higher pSAR to achieve a CV of 0.25 in the heart. This low loss in performance further validates the relative loop phase (0 degrees) implemented in the physical array. Figure 7 demonstrates that the 16LD and 32LD‐SH perform similarly in both the prostate and kidneys. Experimental B_1_^+^ performance in a central 4 × 4 × 4 cm^3^ VOI shows the 32LD‐SH(16Tx) array exhibiting an 18% lower $\eta_{p}$ compared to the 16LD array (Figure S4).

#### 3.2.5 Loading insensitivity

**FIGURE 8:**
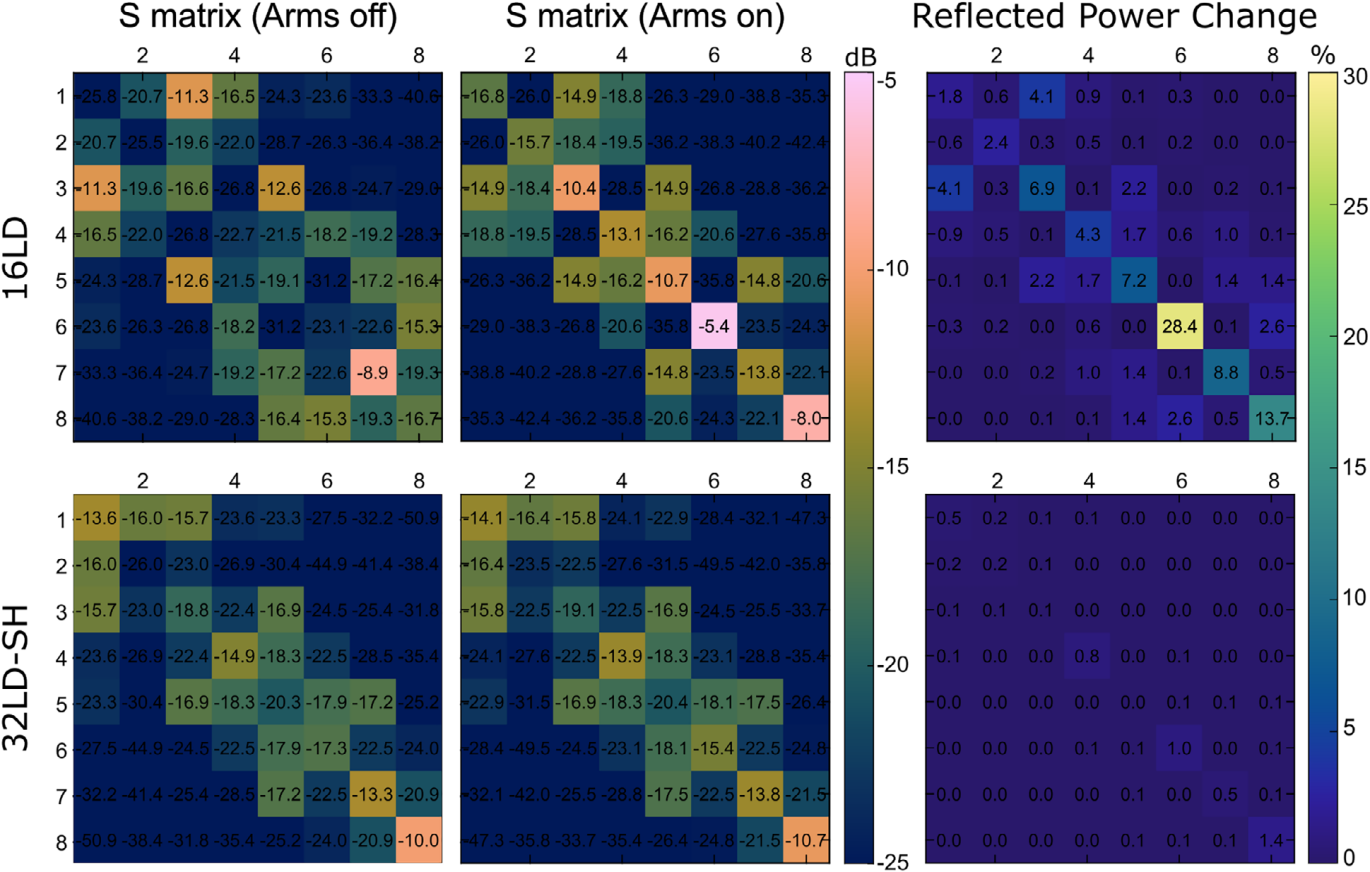
S‐Parameter matrices for the 16LD (top row) and 32LD‐SH (bottom row). When the arms are off the coil (left column), both arrays have reasonable accepted power and coupling. After the arms are placed on top of the arrays (middle column), the matching of the sixth and eighth channels in the 16LD drop significantly. Looking at the change in absolute reflected power (right column) only minute changes were observed in the 32LD‐SH but substantial changes were observed in the 16LD.

The incorporation of an RF shield rendered the 32LD‐SH array substantially less susceptible to top loading compared to the 16LD array. Figure 8 displays S‐parameters of both arrays for two configurations, one with arms positioned laterally and one with the arms atop the arrays. The highest difference in reflected power between the two loading conditions for the 32LD‐SH array was 3%, with all channels maintaining a match better than 10 dB. The 16LD array exhibited a much larger change of 29%, with the match dropping from 23 dB to 5.4 dB when the arms were moved from the subject's side to on top of the array.

#### 3.2.6 Anatomic imaging

**FIGURE 9:**
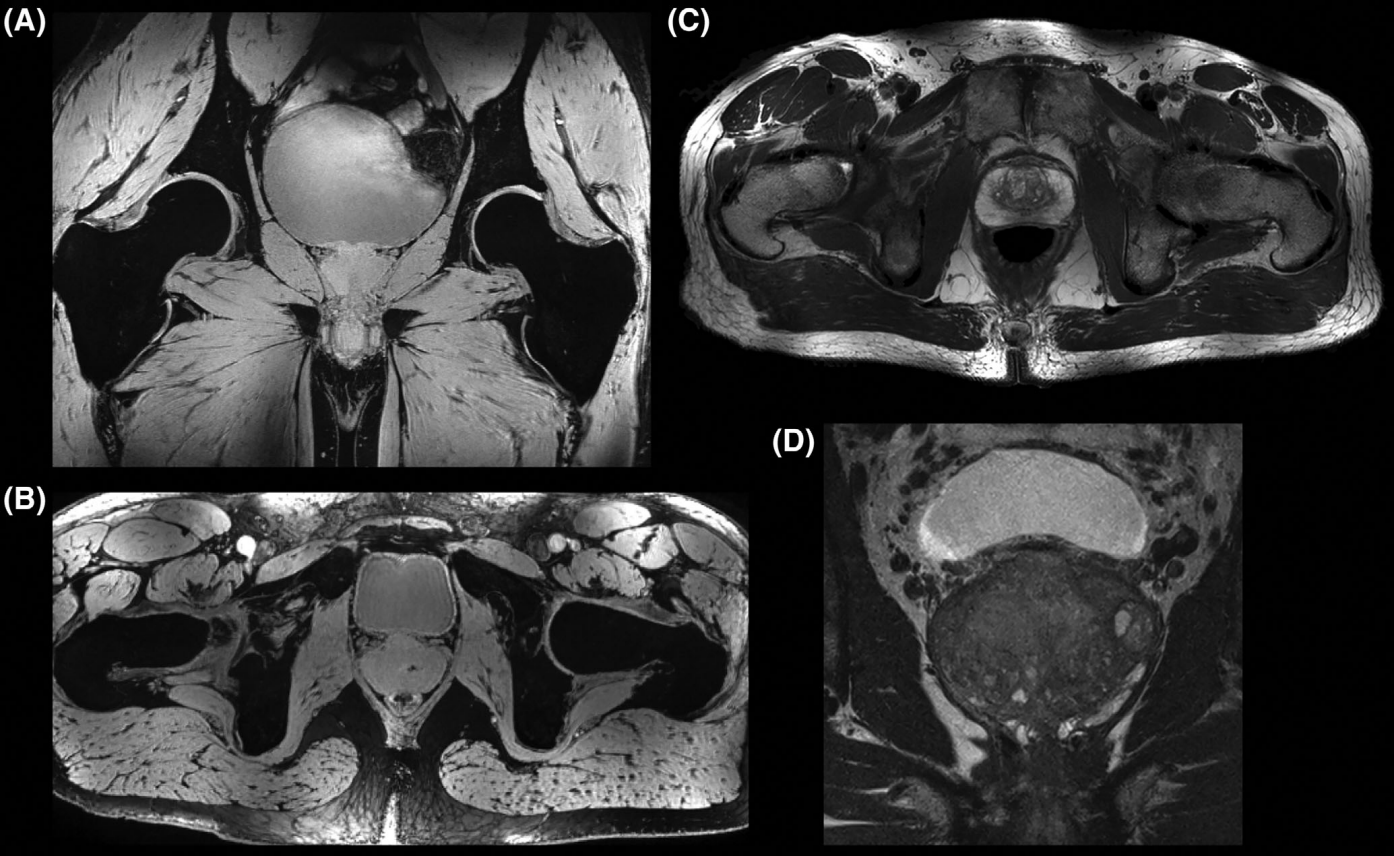
Pelvic imaging. (A) Coronal and (B) axial reformats from a high resolution (0.67 mm isotropic) multi‐echo GRE water‐selective excitation acquired with AMORE. (C) Full FOV anatomic TSE imaging at the level of the prostate using localized AMORE (0.68 × 0.68 × 3 mm^3^). (D) A coronal small FOV TSE anatomic image of the prostate using a localized efficiency shim (resolution 0.45 × 0.45 × 3 mm^3^). Acquisition details are given in Table S1. AMORE, acquisition modes optimized for refocused echoes; GRE, gradient echo; TSE, turbo spin echo.

**FIGURE 10:**
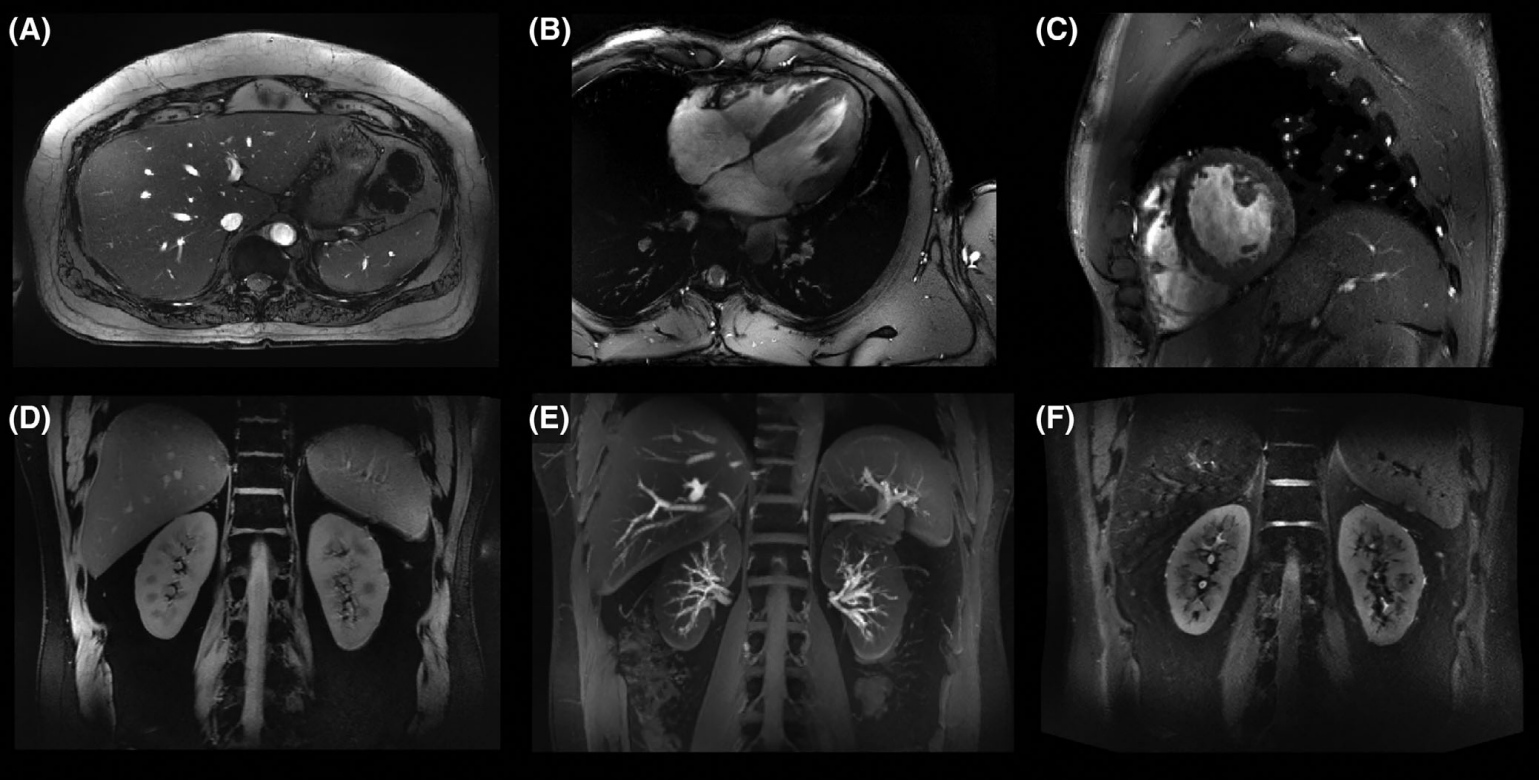
Torso imaging of the liver, kidneys, and heart. Each dataset was acquired during a single breath‐hold with imaging details stated in Table S1. (A) Bias‐corrected anatomic T_1_w imaging at the level of the liver and spleen using TIAMO for RF management. (B) A single frame of a four‐chamber CINE acquired with a CV regularized efficiency shim. (C) A frame of a short‐axis CINE using the same shim as the four‐chamber view. All kidney imaging was acquired using fat suppression and a static efficiency shim over both kidneys and renal arteries, including (D) T_1_w GRE anatomic imaging, (E) multi‐slice in‐flow imaging shown as a 20 mm maximum intensity projection, and (F) TSE imaging showing renal anatomy highlighting the high‐intensity signal from the urine‐filled renal calyces. TIAMO, time interleaved acquisition of modes

Representative images displaying coronal and axial reformats of the fat‐suppressed 3D isotropic GREs of the pelvis acquired with AMORE (Figure 9A,B) demonstrate uniform contrast throughout. The large FOV TSE acquisition at the level of the prostate (Figure 9C) demonstrates the ability to obtain T_2_ weighting while maintaining imaging performance across the entire pelvis. The smaller FOV coronal acquisition of the prostate with a local efficiency shim demonstrates the ability to acquire higher resolution images of deep structures. Outside of the pelvis, similar high‐quality images were obtained in the kidneys, liver, and heart acquired during breath‐holds (Figure 10). The transverse liver image was acquired with AMORE and maintains uniformity in contrast across the entire organ (Figure 10A). Single cardiac phases from the four‐chamber and short‐axis CINE acquisitions show excellent blood pool myocardium contrast as needed for functional imaging. Full cardiac CINE videos are found in supporting videos S1 and  S2. Kidney images (Figure 10D–F) show excellent anaomtic and vascular details, whereas the TSE acquisition demonstrate uniform contrast between the ureter, calyx, pyramids, and blood vessels throughout both kidneys.

## 4 DISCUSSION

In this work, we introduce a 32‐channel transceiver array with a penetrated RF shield and integrated electronics, which exhibits increased receive performance and a significantly reduced sensitivity to top loading. The improved receive performance can be attributed to increased channel count and the positioning of preamplifiers only 2 cm from each element. As designed, all elements are used as transceivers necessitating the use of Tx/Rx switches, which cannot be positioned on‐coil without using a strategy similar to that proposed here. In vivo imaging with the coil demonstrated excellent imaging quality in multiple anatomies throughout the torso.

Because of the geometry of this array, the use of the RF interconnects makes the penetrated RF shield feasible. That is, the motherboard must be pressed onto the coil through the RF shield. If not implemented in a removable fashion, tuning and matching of the elements and maintenance on the final block would not be possible. This type of connection between the resonant elements and electronics minimizes cable exposure to the EM fields, thus alleviating the challenge cable routing. Much effort is spent in coil design to minimize the interaction of direct and RF lines with the transmit fields. Not only can cabling reduce transmit performance,^40^ but it can also complicate the validation process by increasing the error between EM simulations and experimental results because cabling is typically not included in the simulations. Although the impact of the cabling on modeling error has not been evaluated in this work, it is something that can be further explored as we work to establish less restrictive safety factors on the path toward increasing scan efficiency. Furthermore, whereas the RF shield distance to the resonant elements was not exhaustively investigated, the impact of the shield with increasing distance from the elements was explored. The chosen distance of 2 cm resulted in a coil block thickness that is functional for imaging purposes without compromising performance, as demonstrated in the single block simulation and experimental results.

Similar to other higher channel count transceiver arrays, several coil elements share a transmit channel while receiving separately,^41^, ^42^ a strategy that was necessary to accommodate the maximum number of transmit channels available on our pTx system. In general, the implementation of hardware to feed multiple coils has been called *coil compression*.^43^ There are several strategies that seek to optimize this coil compression. Whereas some have tailored an RF shim for the entire array, focusing on a particular anatomy,^44^ we chose to approach the channel combination for a single block with the goal of enabling efficient imaging in more general applications throughout the torso. This was accomplished by exploring what was needed in terms of the amplitude and phase between the three loops to obtain the highest volumetric efficiency when combined with the dipole. The whole volume efficiency optimization in the single block analysis yielded optimal loop phases that were very similar to the 0‐phase implementation. This result supports the use of single‐block, whole volume, optimizations as a future method that can be used to combine any number of transmit channels in an array to match the number of available transmit channels on a given MR system. For example, the compression of this array to eight transmit channels to accommodate the configuration present on most 7 T systems in the field. Additionally, the single‐block optimization method can be expanded to include both magnitude and phase in the shim vector. Although this increases manufacturing complexity, it may be worth the extra design effort to improve performance. A simple tool to aid in the design of an unequal Wilkinson power splitter is given by Sappo et al.^45^ The loss function in the single block optimization can also be changed to maximize different metrics such as $\eta_{\mathrm{sar}}$ or improved homogeneity,^38^ both of which could be achieved by adding a regularization term and adjusting the regularization parameter in the optimization function. It should also be noted that other coil‐combination methods have been reported in the literature, such as the array‐compressed pTx method^43^ and the combination method used in the high‐channel Tic‐Tac‐Toe coil.^41^ Although both of these methods report promising results, we believe that the simplicity of the method outlined here may lead to more consistent performance spanning a wider variety of use cases and therefore deserves its own spot in the MR array designer's toolbox.

When analyzing SAR efficiency, it was found that the pSAR hotspot for the 32LD‐SH array occurred where the loops overlapped (Figure S5). Because our coil design only considered loop coupling, optimizing loop overlap with respect to pSAR would be advantageous and a logical extension of the work. For example, avoiding parallel lines and instead curving the loop traces for lower pSAR, or utilizing a bumped configuration^16^ in these overlapped regions, could be beneficial. Additionally, Figure 7 demonstrates that there are scenarios in which CV can be greatly improved while maintaining a nearly equivalent SAR efficiency. This observation highlights the importance of SAR‐constrained optimizations even when investigating the use of static RF shims. It also demonstrates the utility of adding a CV regularization term when imaging larger or disjoint anatomies such as the heart and kidneys, respectively.

The 32LD‐SH array exhibits superior SNR compared to the reference 16LD array and facilitates parallel imaging acceleration in the previously unavailable foot–head direction. Furthermore, the increase in SNR is consistent with, and further surpasses, a previously presented 24‐channel loop dipole (24LD) array (i.e., eight‐channel dipole transceivers, 16 loop receivers, with two loops per dipole).^46^ The SNR of the 24LD was reported to be 12.5% higher than the SNR of the reference 16LD coil.^20^ For comparison, our 32LD‐SH yielded a 30% central SNR gain compared to the same reference coil. This increase in SNR can be attributed to two factors: (1) the increase in receiver channel count, which is the sole cause of SNR increase from the single‐coil block analysis; and (2) the use of local preamps. Instead of having 4 feet of RG400 cable connecting the 16LD to remote preamps, the 32LD‐SH had preamps directly on‐coil. Using a standard RG400 cable loss of 8.2 dB/100 ft results in a system noise figure that is 93% of the noise figure when there is no cabling before the preamp. Therefore, we estimate that 7% of the SNR increase originates from the local preamp placement. Although not yet explored, the local preamplifier configuration will help facilitate preamp decoupling strategies, which promises to further improve parallel imaging performance. Due to the success of adding additional receiver loops to this array and the success of other high‐channel count receive arrays,^47^, ^48^ future works might consider an even higher channel count receive‐only array, which has the additional benefit of allowing Tx/Rx elements to be optimized independently.

The in‐vivo imaging presented demonstrates functionality in a number of applications. The ability to image local anatomies and full FOV imaging is demonstrated using static or dynamically applied static RF shims, as in the case of AMORE. All images shown here reinforce the premise that proper RF management and hardware development will be able to solve the short wavelength problems associated with UHF. Further experience with the 32LD‐SH will allow us to explore the development of universal shims, previously presented, as viable solutions for other body arrays at 7 T and 10.5 T.^49^ Whereas the double‐oblique imaging in the heart benefitted from parallel imaging in the foot–head direction for the four‐chamber acquisition, we did not fully exploited the acceleration potential of the coil in the current studies. The improved g‐factors in the foot–head direction will benefit other acquisition scenarios, including transverse simultaneous multi‐slice imaging and 3D transverse imaging as used in dynamic contrast and Dixon acquisitions.

## 5 CONCLUSION

A new array consisting of eight dipole and 24 loop elements was constructed and validated for in vivo use. This coil array introduced a penetrated RF shield that enabled on‐coil electronics, made the array insensitive to top loading, and simplified cable management. Furthermore, we show that the RF shield does not significantly impact the fields generated by the loop or dipole elements. Based on the increased channel count and on‐coil electronics, the 32LD‐SH array demonstrated increased SNR with the ability to accelerate image acquisitions in all three orientations. The single‐block optimization method validated the relative loop phase of 0° implemented in the physical array and can be extended to aid in the compression of this array down to an eight‐channel Tx array. Finally, in vivo imaging with this coil demonstrated its potential to unlock the advantages of 7 T for applications throughout the human torso.

## FUNDING INFORMATION

Funding was provided by the National Institutes of Health (NIH), grants NIH P41 EB027061 and NIH R01 EB029985.

## Supporting information


**Table S1.** Acquisition parameters, note that the relative mapping performed for kidney and liver imaging used only 3 slices and a 100ms TR so the acquisition could be performed in a single breath‐hold. flip angle (FA), circular polarized (CP), turbo spin echo (TSE), fat suppressed (FS), actual flip angle imaging (AFI), acquisition modes optimized for refocused echoes (AMORE), parallel imaging reduction/acceleration factor (R), field of view (FOV), echo train length (ETL), readout bandwidth (BW), time of acquisition (TA), signal to noise ratio (SNR).
**Table S2.** A parts list used to create each circuitused in this array.

**Figure S1.** Circuit schematics and close up pictures of the TR switch board and the three‐way Wilkinson power divider.
**Figure S2.** The SOM field (left) of the 3LD‐SH coil block and its MOS field (middle) when applying a phase shim optimized over the entire phantom. The ratio of the two maps (right) demonstrates that the whole volume efficiency shim is able to realize a consistent 80%–85% of the available SOM on the entire left side of the phantom.
**Figure S3.** Axial AFI measurement acquired with each block. For each block an individualized whole volume phase only efficiency shim was calculated and applied to generate the fields shown. The top of the phantom imaging volume is being over flipped leading to the incorrect calculation of lower flip angles on the surface of the phantom.
**Figure S4.** Experimental AFI maps with the new 32LD‐16Tx and the reference 16LD array. Both coils were placed on the same phantom holder and shimmed over an identical ROI, shown in the blue square.
**Figure S5.** Simulated sagittal SAR and B1+ maps demonstrating that the higherst SAR locations occur where the loops are overlapped when different phase sets are applied. The far left is an example of a SAR optimized phase set where the E‐field is more smoothly distributed.

**Video S1.** A 50 phase 4 chamber view CINE of the heart acquired with gating from a pulse oximeter.

**Video S2.** A 50 phase short axis cardiac CINE acquired with gating from a pulse oximeter.
