# Supplementary material for: A shielded 32‐channel body transceiver array with integrated electronics for 7 T
Source: Magn Reson Med. 2025 Mar 30;94(2):852–66. doi: 10.1002/mrm.30498 (PMC12137771; doi:10.1002/mrm.30498)
Supplement: Supplementary file 1 — Table S1. Acquisition parameters, note that the relative mapping performed for kidney and liver imaging used only 3 slices and a 100ms TR so the acquisition could be performed in a single breath‐hold. flip angle (FA), circular polarized (CP), turbo spin echo (TSE), fat suppressed (FS), actual flip angle imaging (AFI), acquisition modes optimized for refocused echoes (AMORE), parallel imaging reduction/acceleration factor (R), field of view (FOV), echo train length (ETL), readout bandwidth (BW), time of acquisition (TA), signal to noise ratio (SNR). Table S2. A parts list used to create each circuitused in this array. [file MRM-94-852-s002.docx]

|  | Relative B­_1_^+^ mapping | Absolute B_1_^+^ mapping | SNR | Full FOV pelvis T2 | Prostate T2 | Anatomic pelvis | Kidney vessels | Anatomic kidney | Kidney T2 | Anatomic liver | Cardiac |
| --- | --- | --- | --- | --- | --- | --- | --- | --- | --- | --- | --- |
| Shimming method | CP | CP | CP | AMORE | Efficiency | AMORE | Efficiency | Efficiency | Efficiency | AMORE | SAR constrained CV |
| Orientation | Transverse | Transverse | Transverse | Transverse | Coronal | Coronal | Coronal | Coronal | Coronal | Transverse | 4-chamber,  Short axis |
| Sequence | 2D axial GRE | 3D axial AFI | 2D axial GRE | 2D TSE | 2D TSE | 3D FS GRE | 2D FS GRE | 2D FS GRE | 2D TSE | 2D GRE | GRE CINE |
| TR (ms) | 500 | 115/15 | 10000 | 3000 | 6000 | 16 | 5.1 | 200 | 3500 | 5.8 | 4.48 |
| TE (ms) | 3.53 | 3.12 | 3.8 | 68 | 85 | 2.47-11.75^†^ | 1.91 | 3.69 | 71 | 2.57 | 2.24 |
| FA (°) | 40 | 60 | 90 | 140 | 120 | 12 | 23 | 17 | 120 | 25 | 18 |
| FOV (mm^2^) | 475 x 238 | 475 x 238 | 475 x 238 | 450x300 | 125 X 121 | 340 x 260 | 400 x 380 | 320 x 336 | 456 x 384 | 380 x 320 | 320 x 270 |
| Slices | 60 | 60 | 60 | 20 | 16 | 128 | 26 | 15 | 3 | 6 | 1 |
| Resolution (mm^3^) | 3.7 x 3.7 x 5 | 3.7 x 3.7 x 5 | 3.7 x 3.7 x 5 | 0.67 x 0.67 x 3 | 0.46 x 0.46 x 3 | 0.67 iso | 1.25 x 1.25 x 3 | 0.8 x 0.8 x 3 | 0.83 x 0.83 x 5 | 0.83 x 0.83 x 5 | 1 x 1 x 4.5 |
| R | - | - | - | 2 | 2 | 6 | 3 | 3 | 2 | 2 | 2 |
| ETL | - | - | - | 11 | 13 | - | - | - | 27 | - |  |
| BW (Hz/Px) | 260 | 490 | 335 | 400 | 385 | 500 | 410 | 210 | 400 | 590 | 505 |
| TA (min) | 0:32*(Nc+2) | 8:19 | 10:40 | 3:06 | 3:37 | 9:55 | 0:26 | 0:27 | 0:17 | 0:27 | 0:16 |
|  |  |  |  |  |  |  |  |  |  |  |  |

TABLE S1: Acquisition parameters, note that the relative mapping performed for kidney and liver imaging used only 3 slices and a 100ms TR so the acquisition could be performed in a single breath-hold.

Abbreviations: flip angle (FA), circular polarized (CP), turbo spin echo (TSE), fat suppressed (FS), actual flip angle imaging (AFI), acquisition modes optimized for refocused echoes (AMORE), parallel imaging reduction/acceleration factor (R), field of view (FOV), echo train length (ETL), readout bandwidth (BW), time of acquisition (TA), signal to noise ratio (SNR).

^†^ Echo times ranged from 2.47 to 11.75 ms in steps of 2.32 ms

| Sub-assembly | Label | Manufacture | Part Number | Value | comments |
| --- | --- | --- | --- | --- | --- |
| Preamp Boards | LNA | Qorvo | QPL9547 |  |  |
|  | VREG | STMicroelectronics | LD2980CM33TR |  |  |
|  | Lc | Coilcraft | 1008CS-122X_R_ | 1200nh |  |
|  | Lom | Coilcraft | 0603CS-56NX_R_ | 56nH |  |
|  | Cb | Knowles-Syfer | 060332000102JXT | 1000pF |  |
|  | R1 |  |  |  |  |
| TR Switches | C1 | ATC | 100B471J500T | 470pF |  |
|  | C2 | ATC | 100B3r9B500T | 3.9pF |  |
|  | C3 | ATC | 100B2r7B500T | 2.7pF |  |
|  | L1 | Coilcraft | 1206CS-122 | 1200nH |  |
|  | L2 | Coilcraft | 1515SQ-82N_E_ | 82nH |  |
|  | Diode | MACOM | MA4P1250NM-1072T |  |  |
|  |  |  |  |  |  |
| Power Divider | C1 | ATC or Knowles Syfer | | 3.1pF |  |
|  | C2 | ATC or Knowles Syfer | | 2.06pF |  |
|  | L1 | Coilcraft | 1111SQ-47N | 47nH |  |
|  | R1 | Vishay Dale Thin Film | PCNM1206E49R9BST5 | 50 Ohm | two in parallel to make a 25 ohm resistor then 6 of these in series to get 150 |

Table S2: A parts list used to create each circuit used in this array.
