# Supplementary material for: A shielded 32‐channel body transceiver array with integrated electronics for 7 T
Source: Magn Reson Med. 2025 Mar 30;94(2):852–66. doi: 10.1002/mrm.30498 (PMC12137771; doi:10.1002/mrm.30498)

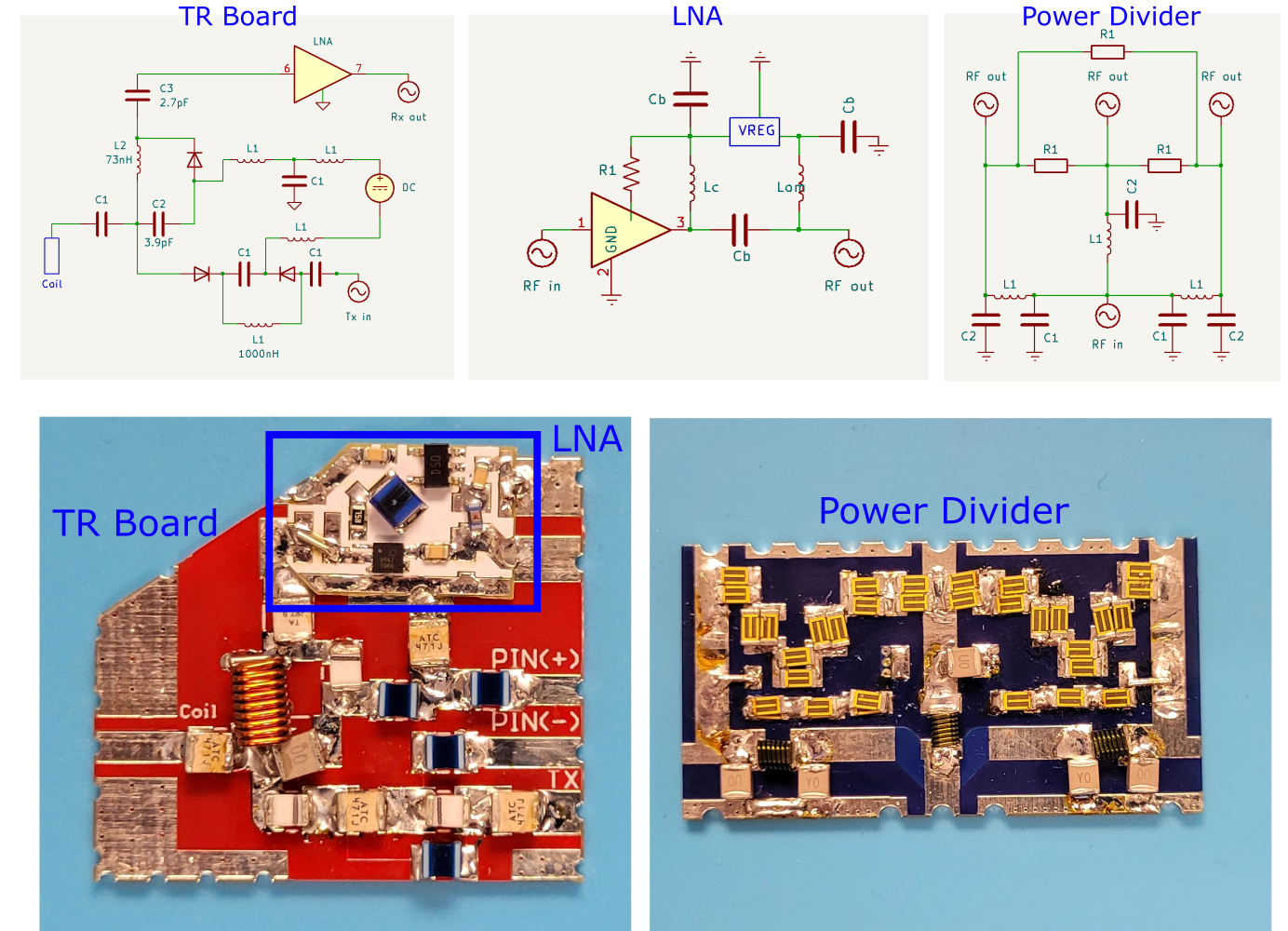


Figure S1: Circuit schematics and close up pictures of the TR switch board and the 3-way Wilkinson power divider.


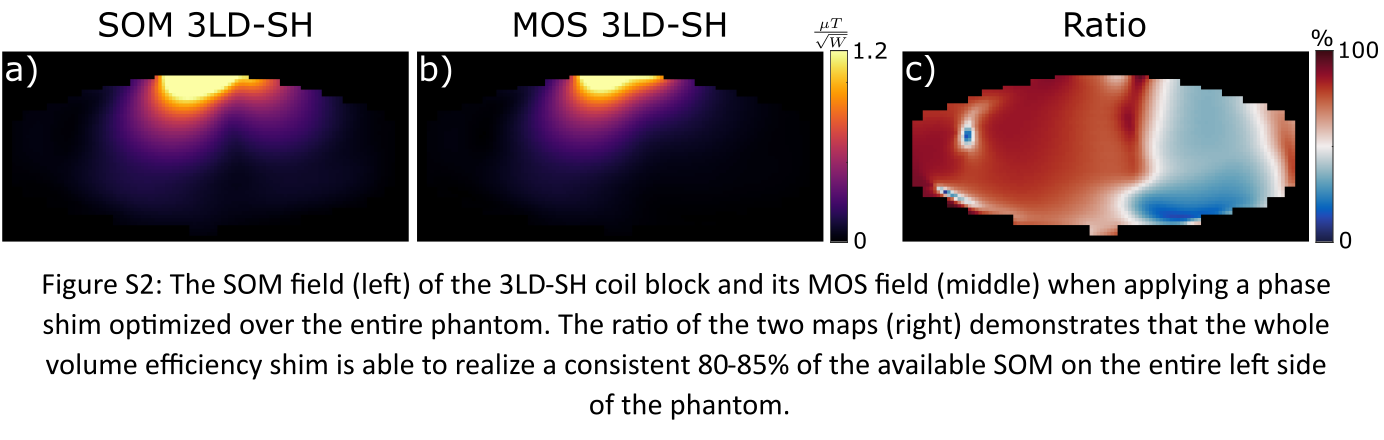


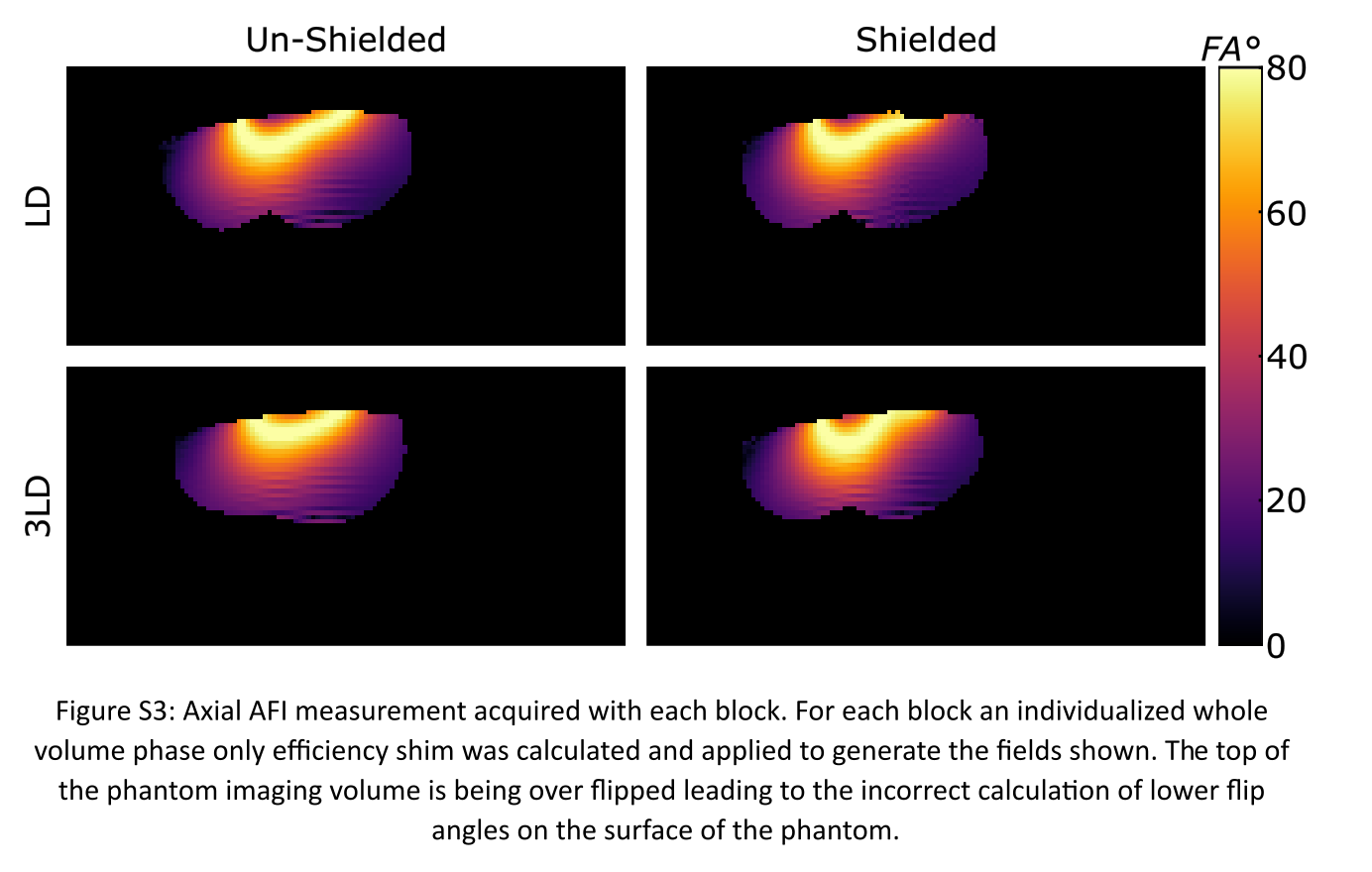


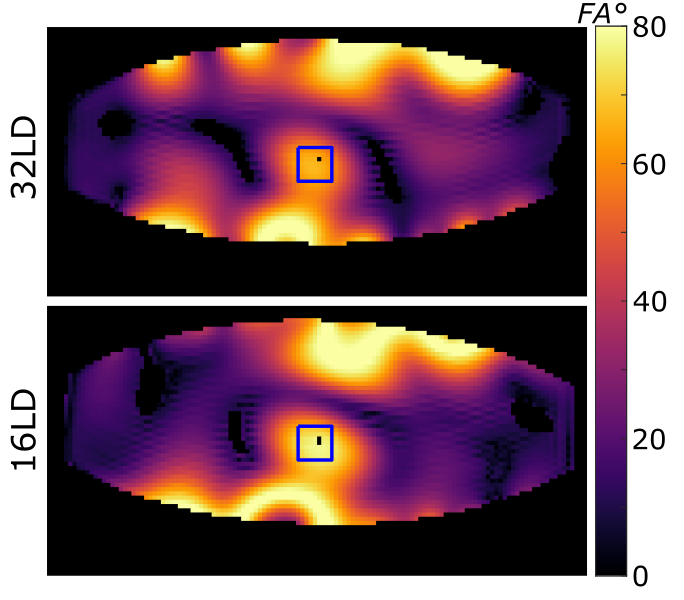


Figure S4: Experimental AFI maps with the new 32LD-16Tx and the reference 16LD array. Both coils were placed on the same phantom holder and shimmed over an identical ROI, shown in the blue square.


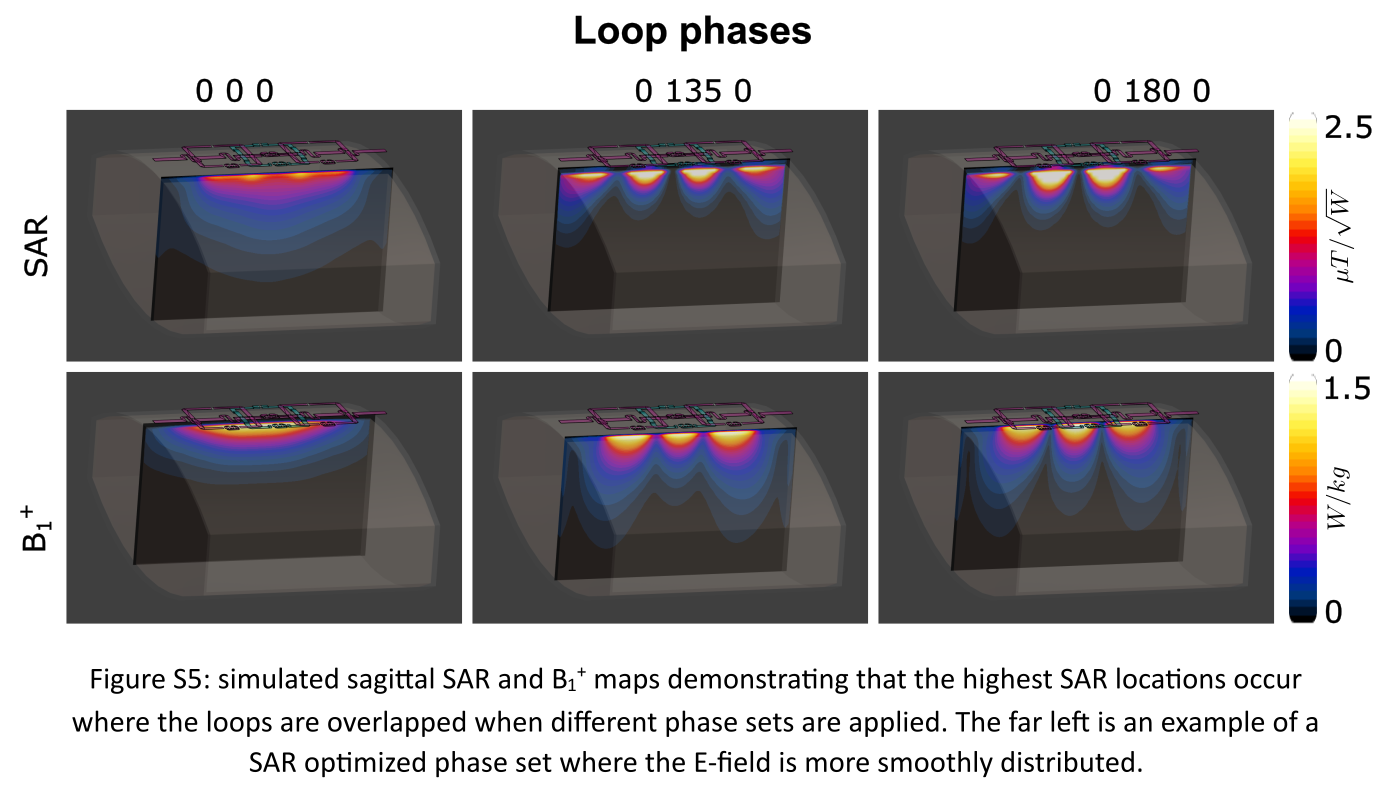

Supplement: Supplementary file 2 — Figure S1. Circuit schematics and close up pictures of the TR switch board and the three‐way Wilkinson power divider. Figure S2. The SOM field (left) of the 3LD‐SH coil block and its MOS field (middle) when applying a phase shim optimized over the entire phantom. The ratio of the two maps (right) demonstrates that the whole volume efficiency shim is able to realize a consistent 80%–85% of the available SOM on the entire left side of the phantom. Figure S3. Axial AFI measurement acquired with each block. For each block an individualized whole volume phase only efficiency shim was calculated and applied to generate the fields shown. The top of the phantom imaging volume is being over flipped leading to the incorrect calculation of lower flip angles on the surface of the phantom. Figure S4. Experimental AFI maps with the new 32LD‐16Tx and the reference 16LD array. Both coils were placed on the same phantom holder and shimmed over an identical ROI, shown in the blue square. Figure S5. Simulated sagittal SAR and B1+ maps demonstrating that the higherst SAR locations occur where the loops are overlapped when different phase sets are applied. The far left is an example of a SAR optimized phase set where the E‐field is more smoothly distributed. [file MRM-94-852-s001.docx]
